# Understanding Physician Work and Well-being Through Social Network Modeling Using Electronic Health Record Data: a Cohort Study

**DOI:** 10.1007/s11606-021-07351-x

**Published:** 2022-01-28

**Authors:** Célia Escribe, Stephanie A. Eisenstat, Kerri Palamara, Walter J. O’Donnell, Jason H. Wasfy, Marcela G. Del Carmen, Sara R. Lehrhoff, Marjory A. Bravard, Retsef Levi

**Affiliations:** 1 Operations Research Center, Massachusetts Institute of Technology, Cambridge, MA USA; 2 Division of General Internal Medicine, Department of Medicine, Massachusetts General Hospital, Boston, MA USA; 3 Harvard Medical School, Boston, MA USA; 4 Pulmonary/Critical Care Division, Massachusetts General Hospital, Boston, MA USA; 5 Cardiology Division, Department of Medicine, Massachusetts General Hospital, Boston, MA USA; 6 Division of Gynecologic Oncology, Massachusetts General Hospital, Boston, MA USA; 7 Massachusetts General Hospital, Boston, MA USA; 8 Sloan School of Management, Massachusetts Institute of Technology, Cambridge, MA USA

**Keywords:** Physician burnout, Physician well-being, EHR, Primary care, Teamwork

## Abstract

**Background:**

Understanding association between factors related to clinical work environment and well-being can inform strategies to improve physicians’ work experience.

**Objective:**

To model and quantify what drivers of work composition, team structure, and dynamics are associated with well-being.

**Design:**

Utilizing social network modeling, this cohort study of physicians in an academic health center examined inbasket messaging data from 2018 to 2019 to identify work composition, team structure, and dynamics features. Indicators from a survey in 2019 were used as dependent variables to identify factors predictive of well-being.

**Participants:**

EHR data available for 188 physicians and their care teams from 18 primary care practices; survey data available for 163/188 physicians.

**Main Measures:**

Area under the receiver operating characteristic curve (AUC) of logistic regression models to predict well-being dependent variables was assessed out-of-sample.

**Key Results:**

The mean AUC of the model for the dependent variables of emotional exhaustion, vigor, and professional fulfillment was, respectively, 0.665 (SD 0.085), 0.700 (SD 0.082), and 0.669 (SD 0.082). Predictors associated with decreased well-being included physician centrality within support team (OR 3.90, 95% CI 1.28–11.97, *P*=0.01) and share of messages related to scheduling (OR 1.10, 95% CI 1.03–1.17, *P*=0.003). Predictors associated with increased well-being included higher number of medical assistants within close support team (OR 0.91, 95% CI 0.83–0.99, *P*=0.05), nurse-centered message writing practices (OR 0.89, 95% CI 0.83–0.95, *P*=0.001), and share of messages related to ambiguous diagnosis (OR 0.92, 95% CI 0.87–0.98, *P*=0.01).

**Conclusions:**

Through integration of EHR data with social network modeling, the analysis highlights new characteristics of care team structure and dynamics that are associated with physician well-being. This quantitative methodology can be utilized to assess in a refined data-driven way the impact of organizational changes to improve well-being through optimizing team dynamics and work composition.

**Supplementary Information:**

The online version contains supplementary material available at 10.1007/s11606-021-07351-x.

## INTRODUCTION

Nearly half of physicians report at least one burnout symptom, including exhaustion, cynicism, and reduced effectiveness.^1–4^ Primary care physicians (PCPs) are especially at high risk of burnout.^1,5^ Physician burnout impacts malpractice claims,^6,7^ turnover,^8–10^ prevalence of substance use disorders,^11,12^ and suicidal ideation.^13,14^ An annual cost of approximately $4.6 billion is attributable to burnout in the USA including resulting physician turnover and reduced clinical hours.^15^

Contributing factors for physician burnout include excessive workload, inefficient practice environments, and electronic health record (EHR) systems.^3,16–19^ While most studies rely on self-reported measures of potential factors (e.g., available time for documentation),^20^ EHR log data has more recently been leveraged to quantify time spent on different tasks,^21,22^ and to find associations between EHR use measures and burnout.^23,24^

While many studies focus on burnout, physician *well-being* is a broader concept,^4,25,26^ encompassing dimensions such as engagement, professional fulfillment, and quality of life dimensions.^27,28^

Interventions targeting work environment structural factors have been shown to reduce physician burnout, suggesting the support care team as an important driver of well-being.^28,29^ A shift from physician-centric to shared-care models could result in improved professional satisfaction.^30,31^ It is also hypothesized that PCPs doing work not requiring physician-level training may impact well-being.^30,32^ Finally, over recent years, EHR inbasket has become a major mode of communication (and work) between PCP, patients, and staff members.^33^ This constitutes a data source to study quantitatively team dynamics in refined ways. Therefore, this study analyzes PCPs’ support team structure and dynamics and the work themes managed in EHR inbasket. To the best of our knowledge, this is the first study to provide a quantitative methodology to describe and relate important aspects of the structure and dynamics of the PCPs’ work environment and well-being.

This study develops a data-driven methodology based on social network modeling and EHR data to capture attributes related to team structure and dynamics and work environment. Social network modeling^34^ has been applied in different fields^35^ to study social structures. Social network graphs consist of *nodes* (individual actors) and *edges* between nodes, typically corresponding to the interaction intensity between two individuals. Leveraging the pairwise intensity of inbasket communications, this approach is applied to the primary care practices to characterize, for each PCP, the respective *support team* with which the PCP works closely, including nurses, medical assistants (MAs), and front-desk staff members (FDs).

Additionally, this study develops analytical models to predict professional well-being metrics including emotional exhaustion, vigor at work, and professional fulfillment. The goal is to identify factors associated with well-being beyond burnout.

## METHODS

### Study Setting and Population

Physicians in an academic medical center (AMC) completed a well-being survey in May 2019 (overall response rate 92%).^36^ Among the respondents, 251 PCPs answered the survey. EHR data was available for 188 PCPs from the AMC and their teams of nurses, MAs, FDs, and other support staff from 18 primary care practices from the AMC from March 1, 2018, through March 1, 2019. All practices from the AMC function independently and have been using the EPIC EHR system since 2016. Survey data was available for 163 out of those 188 physicians (87% response rate).

### Data Sources

The analysis uses a self-constructed dataset linking 4 data sources: (1) EHR inbasket message data; (2) results from the AMC 2019 Physician Survey; (3) PCP patient panel data; and (4) data on work done after standard clinical hours.

The EHR inbasket message database includes all communications between care team members and patients. For each message, the data includes the sender, receiver, the patient ID, and the message text.

The second data source consists of the individual PCP results in the cohort from an internal well-being survey conducted by the AMC and distributed on May 20, 2019. Included were the Maslach Burnout Inventory General Survey (MBI-GS)^37,38^ subscales for emotional exhaustion (7-point frequency, 5-item), cynicism (7-point frequency, 5-item) and personal accomplishment (7-point frequency, 6-item), the Professional Fulfillment Index (PFI)^39^ subscales for professional fulfillment (5-point frequency, 6-item), perceived appreciation (5-point frequency, 6-item) and peer support (5-point frequency, 4-item), and the Utrecht Work Engagement Scale (UWES)^40,41^ subscales for vigor (7-point frequency, 3-item), dedication (7-point frequency, 3-item), and absorption (7-point frequency, 3-item).

The third data source is an internal registry of patient assignments to PCPs’ panel, containing patient risk adjustment scores (Appendix Method 1).

The last data source is the percentage of PCPs’ EHR activity that occurred during non-clinic hours, calculated with an existing methodology (Appendix Method 1).^42^

### Teamwork Analysis Using Social Network Modeling

Social network modeling was used to create the *support team graph* and to analyze quantitatively the characteristics of PCPs’ support team.

#### Methodology to Represent Work Relationships in the PCP Support Team

The first step of the analysis determined the *general practice team graph* for each PCP. All inbasket messages related to the PCPs’ patient panel were considered. Each node in the graph corresponds to a practice member and a patient node represents all PCPs’ panel patients. An edge exists between two nodes in the graph if there is at least one communication documented between the two respective individuals. The *weight* of the edge corresponds to the number of communications.

**Figure 1:**
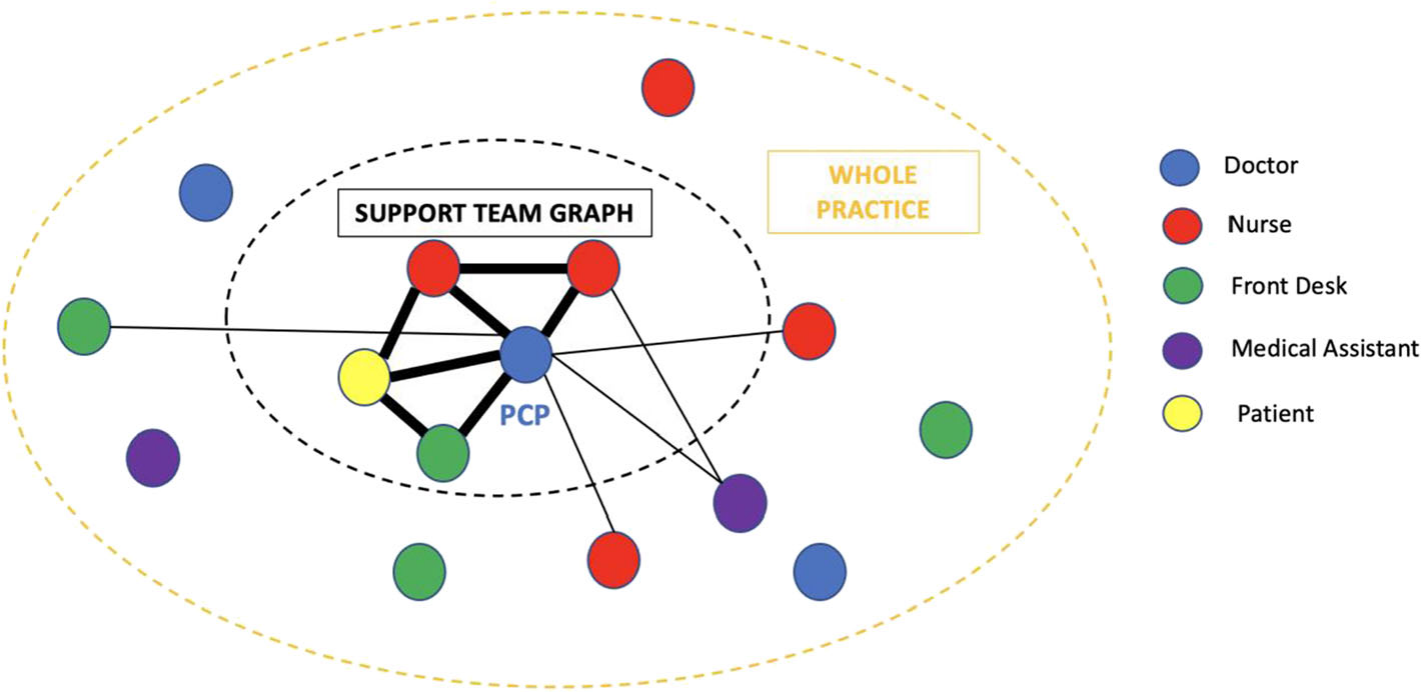
**Schematic representation of PCP support team graph and practice graph. Nodes are staff members where the color represents the team member role. Edges represent inbasket communication between staff members and with patients. The weight of the edge is equal to the number of inbasket communications and is represented by the width of the edge in this representation. The black circle represents the support team graph. It includes all the nodes corresponding to staff members highly involved in communication with the doctor.**

The second step of the analysis created the *support team graph*, made of only team members with whom the PCP interacts the most. Such provider-specific support team graphs are used as most of the network properties heterogeneity between physicians comes from the most significant local working environment. Specifically, the graph is created in the following way (Figure 1). All edges involving the PCP are ordered by decreasing weight. Edges are then selected accordingly to obtain the smallest set that captures at least 60% of the total number of communications involving the PCP. The support team graph then consists of the PCP and the selected nodes with all connecting edges. The 60% threshold was selected by manual review with the help of two physicians. It was further validated by comparing perceived close support team of 20 PCPs with the resulting support team graph (Appendix Method 2).

To capture temporal changes in the support team, the support team graphs were re-calculated for successive periods of 3 months with a rolling 1-month period (e.g., March-May 2018 until January-March 2019). Final features were obtained by calculating the average of a given feature over all time periods.

#### Network-Based Teamwork Features

Teamwork features were calculated based on the support team graph defined above. For a complete list of features and calculation methodology, see Appendix Table 4 and Appendix Method 3, respectively.

To capture the team composition in terms of roles (nurses, MAs, and FDs), the respective proportions of staff members with a specific role within the overall support team graph were calculated as features. The *betweenness centrality* captures the PCPs’ position in the graph, measuring to what extent communication occurs within the support team without involving the PCP. The *entropy feature* of the graph captures the structure of communication within the support team,^43^ and describes how concentrated is the PCPs’ communication with the rest of the support team (e.g., concentration on a few heavy weight edges leads to low entropy, while communication spread evenly across more edges leads to high entropy). *Turnover* within the support team was estimated by comparing the PCPs’ support team from two adjacent time periods and calculating the number of members which left the team.

### Inbasket Work Content Analysis

A descriptive methodology using advanced text analytics^44^ to identify work themes related to inbasket management was developed previously^45^ and was used to derive features corresponding to the share of the PCPs’ inbasket work devoted to specific subcategories (e.g., scheduling). The category *ambiguous diagnosis* corresponds to solving complex diagnosis problems in EHR inbasket. For advanced description of the methodology, refer to Appendix Method 4 and Appendix Table 4.

The ways physicians communicate through inbox can inform team dynamics. The *PCPs’ writing behavior* when writing to patients was captured through the median length of messages written by a PCP to patients. The *nurses’ writing behavior* when writing to the PCP was captured in a similar manner. Any message that was simply forwarded by the nurse to the PCP was considered of length 0; thus, nurses who tend to forward many messages to the PCP will have a lower score.

Finally, control factors were included in the analysis (e.g., gender, years of practice, panel risk score).

### Model Development

#### Dependent Variables

Following existing approaches,^23,36^ the PCPs were classified, based on their score for each of the well-being metrics, into two classes (0 or 1) based on a specified threshold. For example, physicians were classified as 1, i.e., low vigor, or 0, i.e., high vigor. There is heterogeneity in methodology regarding how to choose cutoff points to dichotomize well-being outcomes.^46^ To capture the specific distributions of scores in the studied cohort, the median of the cohort was used as the threshold. Comparison between cohort median and common cutoff points^37,41^ is displayed in Appendix Figure 3. Sensitivity analysis of the predictive models and performance with respect to the threshold was performed with thresholds ranging from 40th % quantile through the median.^46^

#### Predictive Algorithm and Model Selection

Logistic multivariable regression models were implemented.^47,48^ Prior to training, highly correlated independent variables with a Pearson coefficient > 0.75 were removed to reduce multicollinearity. Backward-stepwise feature selection was implemented to include only the most significant features in the model to reduce overfitting and increase the explanation power (Appendix Method 5). Starting with all potential predictors, the feature with the largest *p*-value corresponding to the Z-statistic was sequentially deleted.^49^ The optimal number of features was selected by cross-validation on the out-of-sample AUC.^48,50,51^ The mean out-of-sample AUCs along with associated standard deviation were calculated via 1000 random partitions of the data into 80% training set and 20% held-out set. The cross-validation process allows to measure model performance on a separate dataset, to avoid overestimating the model accuracy. The final model was fitted on the entire training dataset to calculate predictors’ coefficients. Bootstrap resampling was used to validate confidence intervals without data distribution assumptions.

## RESULTS

### Description of Team Dynamics and Variability

Demographic characteristics of the 188 PCPs are displayed in Table 1.

**Table 1:** Cohort Characteristics

**Characteristics**		**Respondents, no. (%)**	**Non-respondents, no. (%)**
**Gender**	**Female**	105 (64)	14 (56)
	**Male**	58 (36)	11 (64)
**Years of practice**	**≤ 9**	19 (12)	8 (32)
	**10–19**	35 (21)	5 (20)
	**20–29**	54 (33)	8 (32)
	**30–39**	39 (24)	3 (12)
	**≥ 40**	14 (9)	1 (4)
	**Unknown**	2 (1)	0 (0)
**Clinical full time equivalent (FTEs)**	**0.90–1.00**	18 (11)	2 (8)
	**0.75–0.89**	18 (11)	3 (12)
	**0.50–0.74**	46 (29)	6 (24)
	**0.25–0.5**	66 (40)	6 (24)
	**< 0.25**	15 (9)	8 (32)
**Clinic type**	**Community health centers**	46 (28)	4 (16)
	**Boston downtown**	117 (72)	21 (84)

Summary statistics for the different features introduced in the “Methods” section are presented in Table 2. Very large standard deviations and ranges were obtained for the different teamwork features. For example, number of nodes 5.63 (SD 2.13, IQ 4.11–6.70), proportion of nurses in support team 44% (SD 13, IQ 36–51%), turnover 14% (SD 10, IQ 0–20%), and betweenness centrality 0.67 (SD 0.32, IQ 0.38–0.86) had the respective mean, standard deviation, and interquantile range (IQ) across PCPs.

**Table 2:** Summary Statistics. The Values Presented in the Table Correspond to the Average of the Different Features Over All the Considered 3-Month Time Periods

**Type of feature**	**Name of feature**	**Mean ± standard deviation (median)**	**Range**	**First quantile**	**Third quantile**
**Structure of support team**	**Number of nodes**	5.63 **±**2.13 (5.10)	2.2–12.6	4.1	6.7
	**Number of edges**	5.82 **±**2.57 (5.35)	1.2–16.7	4.0	7.2
	**Weight**	964.2**±**646.6 (834.8)	73.3–4073.4	483.0	1369.3
	**Proportion of nurses (%)**	44**±**13 (45)	0–79	36	51
	**Proportion of FD (%)**	12**±**12 (8)	0–45	0	21
	**Proportion of MAs (%)**	2**±** 0.6 (0)	0–33	0	0
	**PCP entropy**	1.18**±**0.46 (1.15)	0.05–2.26	0.87	1.48
	**PCP betweenness centrality**	0.67**±**0.32 (0.73)	0–1	0.38	0.86
	**PCP closeness centrality**	159.0**±**138 (140)	8.9–938.9	60.1	201.0
	**Turnover (%)**	14**±**10 (17)	0–33	0	20
**Inbasket work theme allocation**	**Scheduling (%)**	18**±**3 (17)	9–27	15	19
	**Paperwork (%)**	10**±**2 (9)	5–29	8	11
	**Prescription (%)**	5**±**2 (5)	2–10	4	6
	**Administrative referral (%)**	2**±**1 (2)	1–8	1	2
	**Identified symptoms (%)**	7**±**1 (7)	4–13	6	8
	**Ambiguous diagnosis (%)**	13**±**3 (13)	7–22	10	16
	**Condition management (%)**	13**±**2 (14)	8–19	12	15
	**Clinical decision-making referral (%)**	8**±**1 (8)	4–14	7	9
	**Test and exam (%)**	8**±**2 (8)	4–14	7	9
**Inbasket dynamics**	**Length doctor patient (in number of words)**	18.8**±**8.0 (18)	5–47	13.0	22.7
	**Length doctor nurse (in number of words)**	45.2**±**33.9 (40)	0–219	31	51
	**Length nurse doctor (in number of words)**	18.1**±**11.4 (16)	0–65	10	25
**Dependent variables**	**Exhaustion**	17.5**±**7.7 (19)	1–30	11	24
	**Cynicism**	12.1**±**8.0 (10)	0–30	6	18
	**Personal achievement**	27.7**±**6.1 (29)	9–36	24	32
	**Vigor at work**	11.4**±**4.2 (11)	0–18	9	15
	**Dedication**	13.5**±**3.7 (14.5)	0–18	11	16
	**Absorption**	13.4**±**3.7 (14)	0–18	12	16
	**Professional fulfillment**	14.5**±**5.3 (14.5)	0–24	11	19
	**Perceived appreciation**	13.1**±**4.9 (13)	3–24	9.7	17
	**Peer support**	9.8**±**4.0 (10)	0–16	7	12

Features describing inbasket work allocation across subcategories presented smaller standard deviations, but ranges were still large. The relative share of messages handled by PCPs concerned with scheduling and ambiguous diagnosis matters had the respective overall range and interquantile ranges of 9–27% (IQ 15–19%) and 7–22% (IQ: 10–16%).

Statistics were compiled for the 25 PCPs who did not answer the well-being survey. No meaningful difference was observed with the rest of the cohort (Appendix Table 5).

Great variability was observed at the practice level (Appendix Table 6).

### Model Performance for Predicting Well-being

Analysis and discussion of predictive results is limited to the three dependent variables with high AUC scores and giving a good composite picture of well-being—exhaustion, vigor, and professional fulfillment. Predictive performance and selected predictors for the remaining dependent variables are presented in Appendix Table 7 and Appendix Table 8.

**Figure 2:**
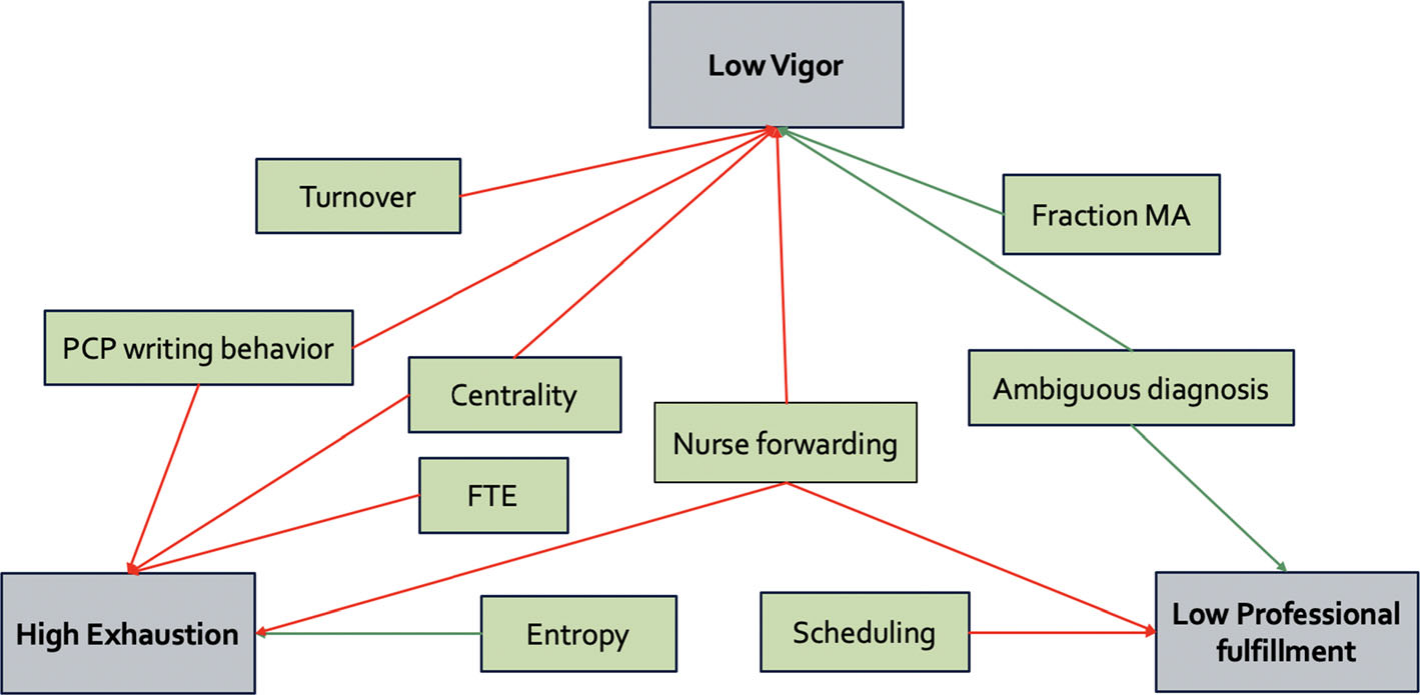
**Selected significant predictors of dependent variables in the logistic regression models. Significant predictors in the logistic regression models are represented here for each of the 3 dependent variables. Green boxes are independent variables, while gray boxes are dependent variables. An arrow indicates when a predictor was significantly associated with a given dependent variable. The color of the arrow indicates if it was correlated with a worsened outcome (red) or with improved outcome (green). The arrows do not represent causal relationships.**

The mean AUC of the multivariable models over the 80%/20% random partitioning for the dependent variables exhaustion, vigor, and professional fulfillment was 0.665 (SD 0.085), 0.700 (SD 0.082), and 0.669 (SD 0.082), respectively. Such AUC values indicate that there is predictive power.^48^ Selected predictors are presented in Table 3 and in Figure 2. Among selected predictors, high physician centrality in the support team was associated with increased exhaustion (OR 4.41, 95% CI 1.05–18.47, *P*=0.04) and decreased vigor (OR 3.90, 95% CI 1.28–11.97, *P*=0.01); increased proportion of messages related to scheduling was associated with decreased professional fulfillment (OR 1.10, 95% CI 1.03–1.17, *P*=0.003), while increased proportion related to ambiguous diagnosis was associated with high vigor and high professional fulfillment (OR 0.92, 95% CI 0.87–0.98, *P*=.01; OR 0.92, 95% CI 0.86–0.99, *P*=0.02); higher fraction of MAs in the support team graph and lower nurses’ forwarding behavior were associated with increased vigor (OR 0.91, 95% CI 0.83–0.99, *P*=0.05); and high PCPs’ FTE was associated with high exhaustion (OR 3.96, 95% CI 1.12–14.03, *P*=0.03), where FTE corresponds to a PCPs’ full-time equivalent workload. Similar confidence intervals were obtained by bootstrap resampling (Appendix Table 9). Exemplar network illustrating dynamics correlated with positive outcomes is displayed in Appendix Figure 4.

**Table 3:** Selected Predictor Variables for Logistic Regression Models

	**MBI exhaustion** Odds ratio (95% CI,*P*value)*	**UWES vigor** **Odds ratio (95% CI,*P*value)**^†^	**Stanford professional fulfillment** **Odds ratio (95% CI,*P*value)**^‡^
**FTE**	3.96 (1.12–14.03, *P*=0.03)		
**Scheduling**			1.10 (1.03–1.17, *P*=0.003)
**Ambiguous diagnosis**		0.92 (0.87–0.98, *P*=0.01)	0.92 (0.86–0.99, *P*=0.02)
**Entropy**	0.24 (0.08–0.70, *P*=0.00)		
**Fraction MA**		0.91 (0.83–0.99, *P*=0.05)	
**Betweenness centrality**	4.41 (1.05–18.47, *P*=0.04)	3.90 (1.28–11.97, *P*=0.01)	
**Turnover**		1.03 (1.00–1.07, *P*=0.07)	
**Length doctor patient**	1.01 (1.00–1.02, *P*=0.05)	1.01 (1.00–1.03, *P*=0.03)	
**Length nurse doctor**	0.93 (0.86–0.99, *P*=0.04)	0.95 (0.92–0.98, *P*=0.008)	0.89 (0.83–0.95, *P*=0.001)

*The outcome 1 corresponds to high exhaustion

†The outcome 1 corresponds to low vigor

‡The outcome 1 corresponds to low professional fulfillment

Sensitivity analysis on the impact of the threshold defining the dependent variables revealed that a choice of threshold ranging from 40 to 50% does not alter the predictive results in any meaningful way (Appendix Tables 10-18).

## DISCUSSION

Team dynamics significantly impact physicians’ well-being. This study aims to provide a quantitative data-driven methodology to analyze team structure and dynamics and their association with PCP well-being, and to measure the impact of specific workflows to inform future areas for improvement.

The obtained features capture the heterogeneity among PCPs related to their respective team structure, including the various interactions among team members, and allow to observe the “real work in real time.” PCPs’ centrality and entropy were shown to vary greatly, highlighting very different internal team dynamics. Variation among PCPs in the share of inbasket work themes suggests that there are differences in work composition, potentially because of how work is shared among team members. Variability across practices suggests practice-level factors impacting support team structure and dynamics. In general, there were relatively few MAs in the support team graph, highlighting that in most clinics included in this study, MAs are not highly involved in inbasket communications with the team, although it is possible that they communicate in-person with other team members.

Different factors were identified as predictors of dependent variables related to PCPs’ well-being, highlighting that well-being is a complex multidimensional concept. High PCP centrality was associated with high exhaustion and low vigor, while high entropy, i.e., PCPs’ communication evenly distributed across many members of the support team, was associated with low exhaustion. This suggests that workflows where communication is spread evenly across support team members, and where staff members coordinate care without directly involving PCPs, are associated with reduced burnout and increased engagement, consistent with the shift to a shared-care model.^30^ Expansion of non-physician staff roles to meet patients’ clinical needs could improve PCP satisfaction.^52^ High fraction of MA’s in the support team graph was found to be associated with high vigor, while increase in nurses’ message forwarding behavior to the PCP was associated with low vigor and low professional fulfillment. This highlights that empowerment of team members impact PCPs’ experience and suggests that there are benefits to including MAs in inbasket communications, and encouraging independence among nurses in managing appropriate inbasket messages from patients.

High support team turnover was found to be associated with low vigor. While it is known that burnout is correlated with higher turnover among PCPs,^8^ this finding indicates that, in addition, turnover within the support team degrades PCPs’ engagement.

Allocation of inbasket work was selected as predictor of vigor and professional fulfillment. Specifically, high share of scheduling work was associated with low professional fulfillment, and a high share of solving complex diagnosis problem was associated with high vigor and high professional fulfillment. This suggests that design of workflow processes that ensure PCPs’ work matches their training level could improve engagement.

Finally, consistent with previous studies,^1^ high FTE was associated with high exhaustion, suggesting that the amount of work hours (e.g., full-time versus part-time) is associated with increasing risk of burnout.

The predictive results and quantitative analysis of practice variability suggest that both the support team structure and dynamics and work content are important predictors of well-being, and that PCPs’ well-being is not merely a function of individual work-style characteristics, but rather the overall surrounding work environment.^4,28,30^ For example, PCPs’ centrality may be influenced by PCPs’ personal style, but more likely is affected primarily by team dynamics.

This predictive model greatly contributes to identifying signs of potential burnout among individual PCPs to be able to intervene early. It should be highlighted however that this work does not identify or quantify the causal impact of different factors on well-being, as well as potential cofounding factors (e.g., communication skills) but rather raises hypotheses with respect to potential such causal mechanisms relying on a data-driven analysis. To prove or disprove these hypotheses will require further research.

Deeper exploration of operational system solutions and potential impact on PCP well-being is necessary. By identifying well-being predictors, this study highlights specific targets for practice and workflow redesign in areas including support staff level, team workflow sharing processes, and inbasket work allocation.

There are limitations to this study. First, while the developed methodology is highly applicable to any medical institution, the model was trained on a relatively small sample of PCPs within an academic medical center where many physicians have other responsibilities than patient care. It would be interesting to observe what similar and new insights are derived from applying this new methodology to PCPs in other settings and more generally physicians from other specialties. Second, while many control factors known to be correlated with physician well-being^16^ are included in the model (e.g., years of practice, gender), other control factors (e.g., presence of social support programs, negative leadership behaviors, relationship status) were not included due to lack of access to relevant data. Third, the small sample size limited the range of predictive models which could be used, as larger sample sizes would allow to use other models such as decision trees to capture nonlinear effects. Fourth, since this analysis relies on inbasket communications, it does not capture additional, potentially important interactions, including face-to-face. Lastly, this analysis focuses on physicians’ well-being. In future work, it is important to obtain and integrate well-being data for the whole care team.

## Supplementary information

ESM 1 (DOCX 54 kb)

## Declarations

This manuscript has not been previously published and is not under consideration in the same or substantially similar form in any other journal. All those listed as authors are qualified for authorship and all qualified to be authors are listed as authors on the byline.

## References

[1] Shanafelt TD, Boone S, Tan L (2012). Burnout and satisfaction with work-life balance among US physicians relative to the general US population. Arch Intern Med..

[2] Shanafelt TD, Hasan O, Dyrbye LN (2015). Changes in Burnout and Satisfaction With Work-Life Balance in Physicians and the General US Working Population Between 2011 and 2014. Mayo Clin Proc..

[3] Shanafelt TD, West CP, Sinsky C (2019). Changes in Burnout and Satisfaction With Work-Life Integration in Physicians and the General US Working Population Between 2011 and 2017. Mayo Clin Proc..

[4] National Academies of Sciences, Engineering, and Medicine; National Academy of Medicine; Committee on Systems Approaches to Improve Patient Care by Supporting Clinician Well-Being. *Taking Action Against Clinician Burnout: A Systems Approach to Professional Well-Being*. National Academies Press (US); 2019. Accessed July 15, 2021. http://www.ncbi.nlm.nih.gov/books/NBK552618/31940160

[5] Bodenheimer T, Chen E, Bennett HD (2009). Confronting the growing burden of chronic disease: can the U.S. health care workforce do the job?. Health Aff (Millwood).

[6] Chen KY, Yang CM, Lien CH (2013). Burnout, job satisfaction, and medical malpractice among physicians. Int J Med Sci..

[7] Balch CM, Oreskovich MR, Dyrbye LN (2011). Personal consequences of malpractice lawsuits on American surgeons. J Am Coll Surg..

[8] Williams ES, Konrad TR, Scheckler WE (2001). Understanding physicians’ intentions to withdraw from practice: the role of job satisfaction, job stress, mental and physical health. Health Care Manage Rev..

[9] Hamidi MS, Bohman B, Sandborg C (2018). Estimating institutional physician turnover attributable to self-reported burnout and associated financial burden: a case study. BMC Health Serv Res..

[10] Willard-Grace R, Knox M, Huang B, Hammer H, Kivlahan C, Grumbach K (2019). Burnout and Health Care Workforce Turnover. Ann Fam Med..

[11] McCain RS, McKinley N, Dempster M, Campbell WJ, Kirk SJ. A study of the relationship between resilience, burnout and coping strategies in doctors. *Postgrad Med J*. Published online August 9, 2017. 10.1136/postgradmedj-2016-134683 10.1136/postgradmedj-2016-134683 28794171

[12] Oreskovich MR, Shanafelt T, Dyrbye LN (2015). The prevalence of substance use disorders in American physicians. Am J Addict..

[13] Dyrbye LN, Thomas MR, Massie FS (2008). Burnout and suicidal ideation among U.S. medical students. Ann Intern Med..

[14] Fridner A, Belkić K, Minucci D (2011). Work environment and recent suicidal thoughts among male university hospital physicians in Sweden and Italy: the health and organization among university hospital physicians in Europe (HOUPE) study. Gend Med..

[15] Han S, Shanafelt TD, Sinsky CA (2019). Estimating the Attributable Cost of Physician Burnout in the United States. Ann Intern Med..

[16] West CP, Dyrbye LN, Shanafelt TD (2018). Physician burnout: contributors, consequences and solutions. J Intern Med..

[17] Melnick ER, Dyrbye LN, Sinsky CA (2020). The Association Between Perceived Electronic Health Record Usability and Professional Burnout Among US Physicians. Mayo Clin Proc..

[18] Shanafelt TD, Sloan JA, Habermann TM (2003). The well-being of physicians. Am J Med..

[19] Freeborn DK (2001). Satisfaction, commitment, and psychological well-being among HMO physicians. West J Med..

[20] Gardner RL, Cooper E, Haskell J (2019). Physician stress and burnout: the impact of health information technology. J Am Med Inform Assoc..

[21] Arndt BG, Beasley JW, Watkinson MD (2017). Tethered to the EHR: Primary Care Physician Workload Assessment Using EHR Event Log Data and Time-Motion Observations. The Annals of Family Medicine..

[22] Tai-Seale M, Olson CW, Li J (2017). Electronic Health Record Logs Indicate That Physicians Split Time Evenly Between Seeing Patients And Desktop Medicine. Health Aff (Millwood)..

[23] Adler-Milstein J, Zhao W, Willard-Grace R, Knox M, Grumbach K (2020). Electronic health records and burnout: Time spent on the electronic health record after hours and message volume associated with exhaustion but not with cynicism among primary care clinicians. J Am Med Inform Assoc..

[24] Tai-Seale M, Dillon EC, Yang Y (2019). Physicians’ Well-Being Linked To In-Basket Messages Generated By Algorithms In Electronic Health Records. Health Aff (Millwood)..

[25] Wallace JE, Lemaire JB, Ghali WA (2009). Physician wellness: a missing quality indicator. Lancet..

[26] Larsen D, Chu JT, Yu L, Chang Y, Donelan K, Palamara K. Correlating Burnout and Well-being in a Multisite Study of Internal Medicine Residents and Faculty. *J Gen Intern Med*. Published online March 5, 2021. 10.1007/s11606-021-06653-4 10.1007/s11606-021-06653-4 PMC8131435 33674923

[27] Konrad TR, Williams ES, Linzer M (1999). Measuring physician job satisfaction in a changing workplace and a challenging environment. SGIM Career Satisfaction Study Group. Society of General Internal Medicine. Med Care..

[28] Shanafelt TD, Noseworthy JH (2017). Executive Leadership and Physician Well-being: Nine Organizational Strategies to Promote Engagement and Reduce Burnout. Mayo Clin Proc..

[29] West CP, Dyrbye LN, Erwin PJ, Shanafelt TD (2016). Interventions to prevent and reduce physician burnout: a systematic review and meta-analysis. Lancet..

[30] Sinsky CA, Willard-Grace R, Schutzbank AM, Sinsky TA, Margolius D, Bodenheimer T (2013). In search of joy in practice: a report of 23 high-functioning primary care practices. Ann Fam Med..

[31] Sinsky CA, Bodenheimer T (2019). Powering-Up Primary Care Teams: Advanced Team Care With In-Room Support. Ann Fam Med..

[32] Altschuler J, Margolius D, Bodenheimer T, Grumbach K (2012). Estimating a reasonable patient panel size for primary care physicians with team-based task delegation. Ann Fam Med..

[33] McMahon LF, Rize K, Irby-Johnson N, Chopra V. Designed to Fail? the Future of Primary Care. *J Gen Intern Med*. Published online July 29, 2020:1-3. 10.1007/s11606-020-06077-6 10.1007/s11606-020-06077-6 PMC7390445 32728962

[34] Scott J (1988). Social Network Analysis. Sociology..

[35] Himelboim I, Smith MA, Rainie L, Shneiderman B, Espina C (2017). Classifying Twitter Topic-Networks Using Social Network Analysis. Social Media + Society.

[36] Rao S, Ferris TG, Hidrue MK (2020). Physician Burnout, Engagement and Career Satisfaction in a Large Academic Medical Practice. Clin Med Res..

[37] Maslach C, Jackson SE, Leiter MP, Schaufeli WB, Schwab RL (1986). *Maslach Burnout Inventory*.

[38] Bakker AB, Demerouti E, Schaufeli WB (2002). Validation of the Maslach Burnout Inventory - General Survey: An Internet Study. Anxiety, Stress, & Coping..

[39] Trockel M, Bohman B, Lesure E (2018). A Brief Instrument to Assess Both Burnout and Professional Fulfillment in Physicians: Reliability and Validity, Including Correlation with Self-Reported Medical Errors, in a Sample of Resident and Practicing Physicians. Academic psychiatry : the journal of the American Association of Directors of Psychiatric Residency Training and the Association for Academic Psychiatry.

[40] Seppälä P, Mauno S, Feldt T (2008). The Construct Validity of the Utrecht Work Engagement Scale: Multisample and Longitudinal Evidence. J Happiness Stud..

[41] Schaufeli W, Bakker AB. *Utrecht Work Engagement Scale: Preliminary Manual*.; 2004. . https://www.wilmarschaufeli.nl/publications/Schaufeli/Test%20Manuals/Test_manual_UWES_English.pdf

[42] Hu M. Leveraging data analytics to improve outpatient healthcare operations. Published online 2020. . https://dspace.mit.edu/handle/1721.1/128043

[43] Dehmer M, Mowshowitz A (2011). A history of graph entropy measures. Information Sciences..

[44] Blei DM, Ng AY, Jordan MI (2003). Latent dirichlet allocation. the Journal of machine Learning research.

[45] Escribe C, Eisenstat S A, O’Donnell WJ, Levi R. How Primary Care Teams and Patients E-Communicate: Identifying Work Themes via Advanced Text Analytics.

[46] Rotenstein LS, Torre M, Ramos MA (2018). Prevalence of Burnout Among Physicians: A Systematic Review. JAMA..

[47] Zemek R, Barrowman N, Freedman SB (2016). Clinical Risk Score for Persistent Postconcussion Symptoms Among Children With Acute Concussion in the ED. JAMA..

[48] Meurer WJ, Tolles J (2017). Logistic Regression Diagnostics: Understanding How Well a Model Predicts Outcomes. JAMA..

[49] Friedman J, Hastie T, Tibshirani R (2001). *The Elements of Statistical Learning*.

[50] Tolles J, Meurer WJ (2016). Logistic Regression: Relating Patient Characteristics to Outcomes. JAMA..

[51] Hanley JA, McNeil BJ (1982). The meaning and use of the area under a receiver operating characteristic (ROC) curve. Radiology..

[52] Shipman SA, Sinsky CA (2013). Expanding primary care capacity by reducing waste and improving the efficiency of care. Health Aff (Millwood)..

