# Supplementary material for: Understanding Physician Work and Well-being Through Social Network Modeling Using Electronic Health Record Data: a Cohort Study
Source: J Gen Intern Med. 2022 Jan 28;37(15):3789–96. doi: 10.1007/s11606-021-07351-x (PMC9640486; doi:10.1007/s11606-021-07351-x)
Supplement: Supplementary file 1 — (DOCX 54 kb) [file 11606_2021_7351_MOESM1_ESM.docx]

**Appendix Method 1: Patient Risk-Adjustement Scores and Methodology for After-Hour Calculation**

Patient Risk Scores

The Health and Human Services-Hierarchical Condition Category, or HHS-HCC, are established by the U.S. Department of Health and Human Services. HHS-HCC scores are federally certified and are designed to predict medical service and drug spending. These scores are primarily driven by diagnosis codes, which are assigned weights according to the severity of the corresponding medical condition.

After-Hour Methodology

An existing methodology was used to determine the percentage of EHR activity during non-clinic hours.^42^ This methodology uses click-level event logging data to measure the time that individual PCPs were actively working in the EHR. Scheduling data was then used to determine individual physician’s clinic hours. This scheduling data was then merged with click-level event logging data to determine the percentage of each physician’s EHR activity that occurred during non-clinic hours. Non-clinic hours include evenings and weekends, as well as normal daytime work hours not included in the physician’s clinic hours that the physicians may have dedicated to other work responsibilities such as research, teaching, and administrative duties.

**Appendix Method 2: Methodology to Select Threshold Defining the Support Team Graph**

When defining the support team graph, all edges involving the PCP are ordered by decreasing weight, and the smallest set of edges in decreasing order containing at least *n*% of the total number of communications of the PCP is selected. All staff members (and patient node) involved in the previously selected edges constituted the PCPs’ support team graph. The *n*% threshold was selected by manual analysis with the help of two physicians. Twenty PCPs among the cohort were selected randomly. Different thresholds ranging from 50 to 80% by 5% steps were used to define the support team graphs. In parallel, the 20 PCPs’ inbox communication with the rest of their team was explored manually with the help of two physicians. Such inbox communications were used to determine which practice team members should be included in the support team graph. Thresholds 50% and 55% led to very large unrealistic support team graphs with too many team members included. Thresholds from 70 to 80% led to too small unrealistic support team graphs which did not include key team members interacting with a given PCP. Thresholds 60% and 65% led to very similar support team graphs which included all team members previously identified by the physicians as members who should be included in the support team graph. The 60% threshold was then selected as the best threshold to define the support team graph by confronting support team graph obtained through this methodology with perceived team graph for 20 PCPs. Those PCPs were asked about their perceived support team graph. This perceived support team graph was confronted to support team graph obtained with the 60% threshold methodology. They were found to match one another, which was not always the case when using the 65% threshold. This validated the choice of the 60% threshold.

**Table 4** Features Included in Machine Learning Model with Corresponding Interpretation

| **Type of feature** | **Feature** | **Interpretation** |
| --- | --- | --- |
| **Teamwork features from social network concepts** | **Number of nodes** | How many staff members are included in the support team |
|  | **Number of edges** | How is communication organized among staff members in the support team |
|  | **Weight** | How much communication happens in the support team |
|  | **Fraction of MAs** | What is the proportion of staff members in the support team with a medical assistant role |
|  | **Fraction of nurses** | What is the proportion of staff members in the support team with a nurse role |
|  | **Fraction of FDs** | What is the proportion of staff members in the support team with an administrative assistant role |
|  | **Ratio patient doctor** | What is the proportion of communication with the patient handled by the PCP |
|  | **PCP betweenness centrality** | How central is the PCP in terms of flow of communication in the support team |
|  | **PCP closeness centrality** | How close is the PCP to all other staff members in the support team in terms of communication |
|  | **PCP entropy** | How concentrated is the PCP’s communication with the rest of the team |
|  | **Turnover** | Fraction of staff members who left the support team between two consecutive time periods |
| **Inbasket work themes features** | **Scheduling** | Share of messages sent and received by PCP concerned with scheduling matters (scheduling appointment, scheduling a call with a patient…) |
|  | **Paperwork** | Share of messages sent and received by PCP concerned with paperwork matters (insurance, health forms, authorizations…) |
|  | **Prescription** | Share of messages sent and received by PCP concerned with administrative prescription matters (refill coordination, problems with pharmacy…) |
|  | **Administrative referral** | Share of messages sent and received by PCP concerned with administrative referrals |
| **Inbasket work themes features** | **Identified symptoms** | Share of messages sent and received by PCP concerned with identified symptoms (acute respiratory problem, musculoskeletal symptom, dermatological problem…) |
|  | **Ambiguous diagnosis** | Share of messages sent and received by PCP concerned with ambiguous diagnosis (ambiguous feelings, ambiguous pain symptoms…) |
|  | **Condition management** | Share of messages sent and received by PCP concerned with condition management (chronic cardiovascular and diabetes condition, medication, nutrition…) |
|  | **Clinical decision-making referral** | Share of messages sent and received by PCP concerned with clinical decision-making referral |
|  | **Test and exam** | Share of messages sent and received by PCP concerned with tests and exams (test result, blood test, imaging exam) |
| **Inbasket writing behavior** | **Length doctor patient** | Median length of messages sent by PCP to patients |
|  | **Length doctor nurse** | Median length of messages sent by PCP to nurses |
|  | **Length nurse doctor** | Median length of messages sent by nurses to PCP |

**Appendix Method 3: Methodology to Calculate Network-Based Teamwork Features**

- The **PCPs’ entropy** is calculated through the following formula:

$$\mathrm{entropy}=-\sum_{e \in edges such that PCP \in e} w_{e}*log(w_{e})$$

where $w_{e}$ is the normalized weight of an edge in the graph. For example, two PCPs connected to 3 staff members with the same global weight of 30 do not have the same entropy: the first PCP with weighted interactions of 28, 1, and 1 has an entropy equal to 0.29, while the second PCP with weighted interactions of 10, 10, and 10 has an entropy of 1.08.

- **Betweenness centrality of a node** in a weighted graph is calculated as follows:

$$betweenness centrality of node v= \sum_{s,t \in V} \frac{\sigma(s,t|v)}{\sigma(s,t)}$$

where $\sigma(s,t)$ is the number of shortest paths between node *s* and node *t*, and where $\sigma(s,t|v)$ is the number of those paths passing through *v*. Here, the algorithm takes into account the distance between two nodes when calculating the shortest paths. This distance is equal to the inverse of the weight of the edge.

- **Closeness centrality of a node** in a weighted graph is calculated as follows:

$$closeness centrality of node v= \frac{n-1}{\sum_{u\in V, u\neq v} d(u,v)}$$

where$d\left( u,v \right)$ is the distance between node *u* and node *v*, and *n* is the number of nodes in the graph. The distance between two nodes is equal to the inverse of the weight of the edge between those two nodes. This feature is only interesting when calculated at the support team graph level. Indeed, if the whole practice graph was used when calculating closeness centrality, low closeness centrality would be obtained for all providers (i.e., doctors’ nodes), as they interact mostly with a small portion of the practice team members (i.e., nodes) corresponding to their close team.

- **Turnover** is calculated by comparing the PCPs’ support team from two adjacent 3-month periods (e.g., March-May 2018 and April-June 2018). The ratio of staff which left the PCPs’ support team between the two periods is calculated. A large ratio value at a given period indicates that practice turnover impacted the considered PCP. The final turnover feature is created by calculating the third quantile of all the turnover estimates over the different time periods for each PCP. The motivation behind using the third quantile instead of the mean or median is to differentiate PCPs who were especially impacted by turnover compared to an average turnover phenomenon affecting all PCPs.

**Appendix Method 4: Methodology to Calculate Inbasket Work Composition Features**

The followed methodology was developed previously.^43^ A Latent Dirichlet Allocation (LDA) model is trained on the inbasket text messages. The LDA model is a generative probabilistic Bayesian model for discovering the topics that occur in a corpus of text documents. The number of desired topics to discover is given as input to the model. After training the model on all text messages, each identified topic is characterized by the respective frequency of each word from the corpus vocabulary. Each message is represented as a weighted mixture of the identified topics. For example, if the LDA model was trained to obtain 3 topics, then a given message can be categorized as the following mixture of topics: 60% topic 1, 40% topic 2, 0% topic 3. Topic 1 is considered the main topic of the message, while topic 3 is not present in the message.

A manual review was then performed to label each selected topic by interpretating of the estimated word frequency within a topic. For example, the first topic identified had the following top ten most frequent words: *Cough, headache, sinus, symptom, allergy, nose, throat, fever, inhaler, infection*. It was therefore labeled “*Upper-Respiratory Symptoms*.”

Topics obtained through the LDA model were grouped into the following categories: *Identified Symptoms, Ambiguous Diagnosis, Condition Management, Tests and Exams, Clinical Decision-Making Referral, Paperwork, Scheduling, Referral, Prescription.*

The share of messages concerned with a given category was then calculated for each PCP, by summing over all messages the respective percentage that this specific topic represented (ranging from 0 to 100%) and then calculating the mean. This corresponds to the features representing inbasket work composition.

**Figure 3 Comparison between median cohort threshold and classical thresholds for dependent variables. We create binary targets by choosing as threshold the cut-off point that separates the dataset into two balanced classes: 50% of the cohort will be classified as 1, and 50% of the cohort will be classified as 0. This threshold is called the median threshold for our cohort. When it is not possible to obtain a perfect balanced dataset because of the well-being score distribution, we choose a threshold in order to get as close as possible to a balanced dataset. We display in red the median threshold which can be compared to classical thresholds in blue.**

**Appendix Method 5: Choice of Predictive Model with Significant Variables Selection**

Using a model relying on all the created covariates leads to high overfitting. Indeed, because of the small size of the cohort (163) and the large number of covariates (27), it is possible to predict almost perfectly the outcome for the train set when using all the covariates. This means that a predictive power close to 100% can be obtained (equivalently, an AUC score close to 1). However, the trained model will perform very poorly on a test set that was not included in the training set. This overfitting takes away the explanation power of the model, which is not generalizable to other settings. The goal of this study is to pick up features which are consistent and stable when explaining well-being among doctors. It is therefore important to obtain a model with strong explanatory power, generalizable to other settings. This explains why a procedure selecting the most significant variables through a predictive model is implemented in this study.

**Table 5** Comparison of Feature Characteristics for PCPs Who Answered the Survey and PCPs Who Did Not Answer the Survey

| **Type of feature** | **Name of feature** | **PCPs who answered the survey (mean ± standard deviation)** | **PCPs who did not answer the survey (mean ± standard deviation)** | ***P* value** |
| --- | --- | --- | --- | --- |
| **Structure of support team** | **Number of nodes** | 5.63 **±**2.13 | 5.84**±**1.66 | 0.56 |
|  | **Number of edges** | 5.82 **±**2.57 | 6.24**±**2.37 | 0.41 |
|  | **Weight** | 964.2 **±**646.6 | 820.6**±**659 | 0.30 |
|  | **Proportion of nurses** | 0.44**±**0.13 | 0.42**±**0.14 | 0.46 |
|  | **Proportion of FD** | 0.12**±**0.12 | 0.12**±**0.11 | 0.87 |
|  | **Proportion of MAs** | 0.02**±**0.06 | 0.05**±**0.10 | 0.10 |
|  | **PCP entropy** | 1.18**±**0.46 | 1.22**±**0.30 | 0.58 |
|  | **PCP betweenness centrality** | 0.67**±**0.32 | 0.65**±**0.25 | 0.43 |
|  | **PCP closeness centrality** | 159.0**±**138 | 114.1**±**112.4 | 0.08 |
|  | **Turnover** | 0.14**±**0.10 | 0.18**±**0.03 | 0.56 |
| **Inbasket work theme allocation** | **Scheduling** | 0.18**±**0.03 | 0.18**±**0.03 | 0.94 |
|  | **Paperwork** | 0.10**±**0.02 | 0.09**±**0.02 | 0.90 |
|  | **Prescription** | 0.05**±**0.02 | 0.05**±**0.02 | 0.31 |
|  | **Administrative referral** | 0.02**±**0.01 | 0.02**±**0.02 | 0.92 |
|  | **Identified symptoms** | 0.07**±**0.01 | 0.06**±**0.02 | 0.04 |
|  | **Ambiguous diagnosis** | 0.13**±**0.03 | 0.14**±**0.03 | 0.12 |
|  | **Condition management** | 0.13**±**0.02 | 0.12**±**0.02 | 0.06 |
|  | **Clinical decision-making referral** | 0.08**±**0.01 | 0.09**±**0.02 | 0.04 |
|  | **Test and exam** | 0.08**±**0.02 | 0.09**±**0.03 | 0.60 |
| **Inbasket dynamics** | **Length doctor patient** | 18.8**±**8.0 | 19.9**±**5.5 | 0.40 |
|  | **Length doctor nurse** | 45.2**±**33.9 | 57.3**±**38.5 | 0.14 |
|  | **Length nurse doctor** | 18.1**±**11.4 | 23.0**±**14.5 | 0.11 |

**Table 6** Features Distribution Across Practices

| **Practice** | **Proportion of nurses** | **Proportion of MAs** | **Proportion of FD** | **Entropy** | **Betweenness** | **Turnover** |
| --- | --- | --- | --- | --- | --- | --- |
| **A** | 0.12 (0.07) | 0.01 (0.02) | 0.43 (0.03) | 0.97 (0.08) | 0.30 (0.17) | 0.09 (0.11) |
| **B** | 0.48 (0.03) | 0.01 (0.03) | 0.06 (0.05) | 1.06 (0.23) | 0.15 (0.14) | 0.06 (0.10) |
| **C** | 0.15 (0.11) | 0.29 (0.03) | 0.00 (0.00) | 0.95 (0.07) | 0.52 (0.34) | 0.16 (0.11) |
| **D** | 0.39 (0.06) | 0.02 (0.03) | 0.38 (0.04) | 1.89 (0.14) | 0.67 (0.10) | 0.19 (0.06) |
| **E** | 0.37 (0.07) | 0.00 (0.01) | 0.27 (0.04) | 1.38 (0.16) | 0.67 (0.18) | 0.13 (0.09) |
| **F** | 0.64 (0.09) | 0.00 (0.00) | 0.07 (0.10) | 1.42 (0.17) | 0.85 (0.07) | 0.19 (0.09) |
| **G** | 0.59 (0.08) | 0.05 (0.06) | 0.12 (0.09) | 1.56 (0.23) | 0.93 (0.06) | 0.20 (0.04) |
| **H** | 0.43 (0.13) | 0.00 (0.00) | 0.21 (0.06) | 1.83 (0.24) | 0.97 (0.04) | 0.16 (0.06) |
| **I** | 0.48 (0.03) | 0.00 (0.00) | 0.00 (0.00) | 0.80 (0.17) | 0.01 (0.02) | 0.09 (0.12) |
| **J** | 0.58 (0.12) | 0.00 (0.00) | 0.11 (0.10) | 1.62 (0.30) | 0.88 (0.07) | 0.12 (0.06) |
| **K** | 0.45 (0.09) | 0.00 (0.00) | 0.02 (0.05) | 0.76 (0.28) | 0.47 (0.30) | 0.08 (0.13) |
| **L** | 0.44 (0.05) | 0.00 (0.00) | 0.04 (0.03) | 0.68 (0.34) | 0.52 (0.26) | 0.17 (0.13) |
| **M** | 0.34 (0.03) | 0.24 (0.03) | 0.05 (0.05) | 1.35 (0.10) | 0.54 (0.23) | 0.09 (0.10) |
| **N** | 0.49 (0.06) | 0.04 (0.07) | 0.09 (0.03) | 1.25 (0.13) | 0.32 (0.21) | 0.18 (0.08) |
| **O** | 0.46 (0.09) | 0.01 (0.02) | 0.15 (0.02) | 1.30 (0.25) | 0.80 (0.11) | 0.20 (0.05) |
| **P** | 0.40 (0.03) | 0.00 (0.00) | 0.25 (0.07) | 1.97 (0.20) | 0.85 (0.04) | 0.17 (0.08) |
| **Q** | 0.39 (0.06) | 0.00 (0.00) | 0.20 (0.05) | 1.16 (0.20) | 0.82 (0.12) | 0.17 (0.08) |

**Table 7** Logistic Regression Prediction Performance for Additional Dependent Variables

| **Dependent Variable** | **Average out-of-sample AUC** | **SD (out-of-sample AUC)** |
| --- | --- | --- |
| **Cynicism** | 0.574 | 0.088 |
| **Personal accomplishment** | 0.700 | 0.084 |
| **Absorption** | 0.610 | 0.088 |
| **Dedication** | 0.691 | 0.084 |
| **Perceived appreciation** | 0.650 | 0.087 |
| **Peer support** | 0.665 | 0.084 |

**Table 8** Selected Predictor Variables for Logistic Regression Model for Additional Dependent Variables

| **Features** | **Cynicism** | **Personal accomplishment** | **Absorption** | **Dedication** | **Perceived appreciation** | **Peer support** |
| --- | --- | --- | --- | --- | --- | --- |
| **Gender** |  |  |  |  |  | 0.30 (0.15–0.60, *P*=0.001) |
| **Years of practice** | 0.98 (0.95–1.00, *P*=0.12) |  |  |  | 0.97 (0.94–1.00, *P*=0.05) |  |
| **After-hour** |  |  | 0.08 (0.01–0.64, *P*=0.01) |  |  |  |
| **Risk score** | 1.11 (0.94–1.31, *P*=0.18) |  |  |  | 1.65 (1.18–2.31, *P*=0.003) |  |
| **Scheduling** |  | 1.08 (1.00–1.17, *P*=0.05) | 1.11 (1.04–1.19, *P*=0.002) | 1.13 (1.05–1.22, *P*=0.00) |  |  |
| **Paperwork** |  | 1.17 (1.01–1.36, *P*=0.03) |  |  |  | 1.11 (1.04–1.17, *P*=0.001) |
| **Ambiguous diagnosis** |  |  |  |  | 0.90 (0.83–0.98, *P*=0.01) |  |
| **Fraction MA** |  | 0.93 (0.87–0.99, *P*=0.04) |  | 0.94 (0.89–0.99, *P*=0.05) | 0.93 (0.87–1.00, *P*=0.09) | 0.93 (0.87–1.00, *P*=0.06) |
| **Fraction nurse** |  | 0.95 (0.93–0.98, *P*=0.001) |  | 0.97 (0.94–0.99, *P*=0.006) |  |  |
| **Length nurse doctor** |  | 0.95 (0.92–0.98, *P*=0.005) | 0.97 (0.94–0.99, *P*=0.04) | 0.88 (0.83–0.95, *P*=0.001) |  |  |

**Table 9** Selected Predictor Variables for Logistic Regression Model with Bootstrap Resampling with 1000 samples

|  | **MBI exhaustion, odds ratio (95% CI)** | **UWES vigor, odds ratio (95% CI)** | **Stanford professional fulfillment, odds ratio (95% CI)** |
| --- | --- | --- | --- |
| **FTE** | 4.17 (1.51–12.88) |  |  |
| **Scheduling** |  |  | 1.10 (1.04–1.16) |
| **Ambiguous diagnosis** |  | 0.91 (0.86–0.96) | 0.92 (0.86–0.98) |
| **Entropy** | 0.23 (0.08–0.56) |  |  |
| **Fraction MA** |  | 0.88 (0.81–0.97) |  |
| **Betweenness centrality** | 4.72 (1.42–17.89) | 4.15 (1.49–12.45) |  |
| **Turnover** |  | 1.03 (1.00–1.07) |  |
| **Length doctor patient** | 1.01 (1.00–1.02) | 1.01 (1.00–1.03) |  |
| **Length nurse doctor** | 0.93 (0.87–0.97) | 0.95 (0.91–0.98) | 0.89 (0.83–0.94) |

**Figure 4 Exemplar network. Exemplar network illustrating dynamics correlated with positive outcomes. In this network, the doctor’s node has high entropy (communication spread evenly across many edges) and low betweenness centrality (communication can often occur within the support team without involving the PCP). The fraction of MAs in this network is high. Blue node corresponds to the doctor, red nodes correspond to nurses, green nodes correspond to administrative staff, purple nodes correspond to MAs, and yellow node corresponds to the patient node.**

**Table 10** Sensitivity to the Choice of the Threshold Used to Define Emotional Exhaustion Binary Outcome

|  | **Threshold 20* (43%**^†^**)**  **Odds ratio (95% CI, *P* value)** | **Threshold 19* (50%**^†^**)**  **Odds ratio (95% CI, *P* value)** |
| --- | --- | --- |
| **Out-of-sample** **AUC score** | 0.675 (SD 0.082) | 0.665 (SD 0.085) |
|  | | |
| **FTE** | 3.99 (1.15–13.85, *P*=0.03) | 3.96 (1.12–14.03, *P*=0.03) |
| **Entropy** | 0.14 (0.05–0.41, *P*=0.00) | 0.24 (0.08–0.70, *P*=0.00) |
| **Betweenness centrality** | 3.71 (0.93–14.80, *P*=0.06) | 4.41 (1.05–18.47, *P*=0.04) |
| **Length doctor patient** | 1.01 (1.00–1.02, *P*=0.02) | 1.01 (1.00–1.02, *P*=0.05) |
| **Length nurse doctor** |  | 0.93 (0.86–0.99, *P*=0.04) |

*The indicated threshold *t* (e.g., 20) is used to create a binary dependent variable. An emotional exhaustion score higher or equal to *t* is translated into a binary score of 1

^†^The indicated percentage corresponds to the final proportion of the cohort classified as 1

**Table 11** Sensitivity to the Choice of the Threshold Used to Define Vigor Binary Outcome

|  | **Threshold 3.00* (33%**^†^**)**  **Odds ratio (95% CI, *P* value)** | **Threshold 3.33* (45%**^†^**)**  **Odds ratio (95% CI, *P* value)** | **Threshold 3.66* (55%**^†^**)**  **Odds ratio (95% CI, *P* value)** |
| --- | --- | --- | --- |
| **Out-of-sample** **AUC score** | 0.669 (SD 0.086) | 0.700 (SD 0.082) | 0.675 (SD 0.087) |
|  |  |  |  |
| **Ambiguous diagnosis** | 0.95 (0.91–1.00, *P*=0.05) | 0.92 (0.87–0.98, *P*=0.01) | 0.95 (0.89–1.00, *P*=0.08) |
| **Fraction MA** | 0.90 (0.80–1.00, *P*=0.07) | 0.91 (0.83–0.99, *P*=0.05) | 0.93 (0.87–1.00, *P*=0.06) |
| **Betweenness centrality** |  | 3.90 (1.28–11.97, *P*=0.01) | 5.66 (2.08–15.37, *P*=0.001) |
| **Turnover** | 1.05 (1.01–1.08, *P*=.004) | 1.03 (1.00–1.07, *P*=.07) |  |
| **Length doctor patient** |  | 1.01 (1.00–1.03, *P*=.03) | 1.01 (1.00–1.02, *P*=.10) |
| **Length nurse doctor** | 0.96 (0.93–0.99, *P*=.02) | 0.95 (0.92–0.98, *P*=.008) | 0.97 (0.93–1.00, *P*=.05) |

*The indicated threshold t (e.g., 3.00) is used to create a binary dependent variable. A vigor score lower or equal to t is translated into a binary score of 1

^†^The indicated percentage corresponds to the final proportion of the cohort classified as 1

**Table 12** Sensitivity to the Choice of the Threshold Used to Define Professional Fulfillment Binary Outcome

|  | **Threshold 13* (44%**^†^**)**  **Odds ratio (95% CI, *P* value)** | **Threshold 14* (50%**^†^**)**  **Odds ratio (95% CI, *P* value)** |
| --- | --- | --- |
| **Out-of-sample** **AUC score** | 0.628 (0.08) | 0.669 (SD 0.082) |
|  |  |  |
| **Scheduling** | 1.07 (1.01–1.14, *P*=0.01) | 1.10 (1.03–1.17, *P*=0.003) |
| **Ambiguous diagnosis** | 0.92 (0.86–0.99, *P*=0.03) | 0.92 (0.86–0.99, *P*=0.02) |
| **Length nurse doctor** | 0.91 (0.86–0.98, *P*=0.009) | 0.89 (0.83–0.95, *P*=0.001) |

*The indicated threshold *t* (e.g., 13) is used to create a binary dependent variable. A professional fulfillment score lower or equal to *t* is translated into a binary score of 1

^†^The indicated percentage corresponds to the final proportion of the cohort classified as 1

**Table 13** Sensitivity to the Choice of the Threshold Used to Define Cynicism Binary Outcome

|  | **Threshold 13* (43%**^†^**)**  **Odds ratio (95% CI, *P* value)** | **Threshold 12* (46%**^†^**)**  **Odds ratio (95% CI, *P* value)** | **Threshold 11* (48%**^†^**)**  **Odds ratio (95% CI, *P* value)** |
| --- | --- | --- | --- |
| **Out-of-sample** **AUC score** | 0.572 (0.085) | 0.563 (SD 0.090) | 0.574 (SD 0.088) |
|  |  |  |  |
| **FTE** |  |  |  |
| **Years of practice** | 0.98 (0.97–0.99, *P*=0.02) | 0.99 (0.97–1.00, *P*=0.10) | 0.98 (0.95–1.00, *P*=0.12) |
| **Risk score** |  |  | 1.11 (0.94–1.31, *P*=0.18) |

*The indicated threshold *t* (e.g., 13) is used to create a binary dependent variable. A cynicism score higher or equal to t is translated into a binary score of 1

^†^The indicated percentage corresponds to the final proportion of the cohort classified as 1

**Table 14** Sensitivity to the Choice of the Threshold Used to Define Personal Accomplishment Binary Outcome

|  | **Threshold 27* (42%**^†^**)**  **Odds ratio (95% CI, *P* value)** | **Threshold 28* (48%**^†^**)**  **Odds ratio (95% CI, *P* value)** |
| --- | --- | --- |
| **Out-of-sample** **AUC score** | 0.681 (0.084) | 0.700 (SD 0.084) |
|  |  |  |
| **Scheduling** |  | 1.08 (1.00–1.17, *P*=0.05) |
| **Paperwork** | 1.14 (1.00–1.30, *P*=0.04) | 1.17 (1.01–1.36, *P*=0.03) |
| **Fraction MA** | 0.92 (0.85–0.99, *P*=0.03) | 0.93 (0.87–0.99, *P*=0.04) |
| **Fraction nurse** | 0.96 (0.94–0.98, *P*=0.003) | 0.95 (0.93–0.98, *P*=0.001) |
| **Length nurse doctor** | 0.96 (0.92–0.99, *P*=0.02) | 0.95 (0.92–0.98, *P*=0.005) |
| **Betweenness centrality** | 3.78 (1.11–12.89, *P*=0.03) |  |

*The indicated threshold t (e.g., 27) is used to create a binary dependent variable. A personal accomplishment score lower or equal to t is translated into a binary score of 1

^†^The indicated percentage corresponds to the final proportion of the cohort classified as 1

**Table 15** Sensitivity to the Choice of the Threshold Used to Define Dedication Binary Outcome

|  | **Threshold* 4.33 (40%**^†^**)**  **Odds ratio (95% CI, *P* value)** | **Threshold* 4.66 (50%**^†^**)**  **Odds ratio (95% CI, *P* value)** |
| --- | --- | --- |
| **Out-of-Sample** **AUC score** | 0.653 (0.087) | 0.691 (SD 0.084) |
|  |  |  |
| **Scheduling** | 1.08 (1.02–1.16, *P*=0.01) | 1.13 (1.05–1.22, *P*=0.00) |
| **Fraction MA** |  | 0.94 (0.89–0.99, *P*=0.05) |
| **Fraction nurse** | 0.97 (0.94–0.99, *P*=0.007) | 0.97 (0.94–0.99, *P*=0.006) |
| **Length nurse doctor** | 0.90 (0.84–0.97, *P*=0.005) | 0.88 (0.83–0.95, *P*=0.001) |

*The indicated threshold *t* (e.g., 4.33) is used to create a binary dependent variable. A dedication score lower or equal to *t* is translated into a binary score of 1

^†^The indicated percentage corresponds to the final proportion of the cohort classified as 1

**Table 16** Sensitivity to the Choice of the Threshold Used to Define Absorption Binary Outcome

|  | **Threshold* 4.33 (44%**^†^**)**  **Odds ratio (95% CI, *P* value)** | **Threshold* 4.66 (54%**^†^**)**  **Odds ratio (95% CI, *P* value)** |
| --- | --- | --- |
| **Out-of-sample** **AUC score** | 0.652 (0.085) | 0.610 (SD 0.088) |
|  |  |  |
| **After-hour** | 0.01 (0.00–0.22, *P*=0.002) | 0.08 (0.01–0.64, *P*=0.01) |
| **Risk score** | 1.25 (1.00–1.56, *P*=0.04) |  |
| **Scheduling** | 1.09 (1.02–1.17, *P*=0.009) | 1.11 (1.04–1.19, *P*=0.002) |
| **Length nurse doctor** | 0.95 (0.92–0.98, *P*=0.004) | 0.97 (0.94–0.99, *P*=0.04) |

*The indicated threshold *t* (e.g., 4.33) is used to create a binary dependent variable. An absorption score lower or equal to t is translated into a binary score of 1

^†^The indicated percentage corresponds to the final proportion of the cohort classified as 1

**Table 17** Sensitivity to the Choice of the Threshold Used to Define Perceived Appreciation Binary Outcome

|  | **Threshold* 12 (47%**^†^**)**  **Odds ratio (95% CI, *P* value)** | **Threshold* 13 (55%**^†^**)**  **Odds ratio (95% CI, *P* value)** |
| --- | --- | --- |
| **Out-of-sample** **AUC score** | 0.650 (SD 0.087) | 0.685 (SD 0.086) |
|  |  |  |
| **Years of practice** | 0.97 (0.94–1.00, *P*=0.05) | 0.96 (0.22–0.99, *P*=0.02) |
| **Risk score** | 1.65 (1.18–2.31, *P*=0.003) | 1.69 (1.18–2.42, *P*=0.004) |
| **Ambiguous diagnosis** | 0.90 (0.83–0.98, *P*=0.01) | 0.91 (0.84–0.99, *P*=0.04) |
| **Paperwork** |  | 1.14 (1.02–1.27, *P*=0.01) |
| **Fraction MA** | 0.93 (0.87–1.00, *P*=0.09) | 0.92 (0.84–1.00, *P*=0.06) |
| **Length nurse doctor** |  | 0.95 (0.92–0.98, *P*=0.01) |

*The indicated threshold *t* (e.g., 12) is used to create a binary dependent variable. A perceived appreciation score lower or equal to t is translated into a binary score of 1

^†^The indicated percentage corresponds to the final proportion of the cohort classified as 1

**Table 18** Sensitivity to the Choice of the Threshold Used to Define Peer Support Binary Outcome

|  | **Threshold* 9 (45%**^†^**)**  **Odds ratio (95% CI, *P* value)** | **Threshold* 10 (51%**^†^**)**  **Odds ratio (95% CI, *P* value)** |
| --- | --- | --- |
| **Out-of-sample** **AUC score** | 0.653 (SD 0.083) | 0.665 (SD 0.084) |
|  |  |  |
| **Gender** | 0.38 (0.19–0.77, *P*=0.007) | 0.30 (0.15–0.60, *P*=0.001) |
| **Paperwork** | 1.15 (1.04–1.27, *P*=0.004) | 1.11 (1.04–1.17, *P*=0.001) |
| **Ambiguous diagnosis** | 0.92 (0.86–0.99, *P*=0.02) |  |
| **Fraction MA** |  | 0.93 (0.87–1.00, *P*=0.06) |

*The indicated threshold *t* (e.g., 9) is used to create a binary dependent variable. A peer support score lower or equal to *t* is translated into a binary score of 1

^†^The indicated percentage corresponds to the final proportion of the cohort classified as 1
